# A Variable Horizon Model Predictive Control for Magnetorheological Semi-Active Suspension with Air Springs

**DOI:** 10.3390/s24216926

**Published:** 2024-10-29

**Authors:** Gang Li, Lin Zhong, Wenjun Sun, Shaohua Zhang, Qianjie Liu, Qingsheng Huang, Guoliang Hu

**Affiliations:** 1 Key Laboratory of Vehicle Intelligent Equipment and Control of Nanchang City, East China Jiaotong University, Nanchang 330013, China; 2 Key Laboratory of Conveyance and Equipment, Ministry of Education, East China Jiaotong University, Nanchang 330013, China; 3 Yingtan Applied Engineering School, Yingtan 335211, China

**Keywords:** magnetorheological damper, semi active suspension, model predictive control, variable horizon, air spring

## Abstract

To improve the characteristics of traditional model predictive control (MPC) semi-active suspension that cannot achieve the optimal suspension control effect under different conditions, a variable horizon model predictive control (VHMPC) method is devised for magnetorheological semi-active suspension with air springs. Mathematical models are established for the magnetorheological dampers and air springs. Based on the improved hyperbolic tangent model, a forward model is established for the magnetorheological damper. The adaptive fuzzy neural network method is used to establish the inverse model of the magnetorheological damper. The relationship between different road excitation frequencies and the control effect of magnetorheological semi-active suspension with air springs is simulated, and the optimal prediction horizons under different conditions are obtained. The VHMPC method is designed to automatically switch the predictive horizon according to the road surface excitation frequency. The results demonstrate that under mixed conditions, compared with the traditional MPC, the VHMPC can improve the smoothness of the suspension by 2.614% and reduce the positive and negative peaks of the vertical vibration acceleration by 11.849% and 6.938%, respectively. Under variable speed road conditions, VHMPC improved the sprung mass acceleration, dynamic tire deformation, and suspension deflection by 7.191%, 7.936%, and 22.222%, respectively, compared to MPC.

## 1. Introduction

Suspension systems have a crucial impact on vehicle handling stability, driving safety, and ride comfort [1]. Depending on the form of control, suspension systems are categorized into three main types: passive suspension, active suspension, and semi-active suspension [2]. Passive suspensions are unable to adapt to changes in body parameters and driving conditions because the damping and stiffness coefficients are fixed to certain values. Active suspensions rely on the actuator to apply active force to the suspension, and although they realize superior suspension performance, they have the disadvantages of complex structure and high energy consumption. The variable damper in semi-active suspension can provide passive damping force to the suspension. Under the control of appropriate intelligent control algorithms, the vehicle suspension system can have both better maneuverability and comfort. Therefore, it has become a research hotspot [3,4,5].

The types of dampers for semi-active suspensions mainly include solenoid valve dampers and magnetorheological dampers (MRD). Different from the complex structure of the solenoid valve damper, the magnetorheological damper is characterized by a simple structure and rapid response. The magnetorheological damper is a new type of damper based on the magnetorheological effect of the magnetorheological fluid. By applying different levels of excitation current to the excitation coil in the magnetorheological damper, the excitation coil generates different strengths of the magnetic field, which changes the yield strength of the magnetorheological fluid under the magnetorheological effect and generates different resistances so that the magnetorheological damper is capable of outputting continuously adjustable damping force. Magnetorheological dampers have the advantages of a simple structure, a large output force, low energy consumption, a fast response speed (milliseconds), and a continuously adjustable damping force, and they are easy to combine with microcomputer control in engineering applications. They have a wide range of applications in civil engineering, machinery, and aviation fields [6,7,8].

The commonly used control methods for semi-active suspension include skyhook control, groundhook control, PID control, fuzzy control, sliding mode control, etc. However, these control methods generally have the disadvantages of insufficient robustness or over-reliance on expert experience [9,10,11,12]. Furthermore, most semi-active suspension control methods require the high accuracy of mathematical models [13]. In the process of practical application, we cannot establish a high-precision mathematical model of the controlled object, so the actual control effect is slightly inferior to the theoretical simulation results. MPC algorithms have the advantages of not requiring the high accuracy of mathematical models, good tracking performance, and robustness to model errors, and the application of MPC algorithms in the field of vehicle suspension has become a hot research topic [14]. Mai N V et al. [15] investigated the application of MPC for vibration control in a quarter-suspension system. The model predictive control was designed to deal with control input constraints, and multi-parameter planning was used to compute the explicit solution of model predictive control to achieve a reduction in computation time in practical applications. The results show that the dynamic travel of the suspension is substantially reduced compared to the passive suspension. Theunissen J et al. [16] solved the optimization problem offline through multi-parameter quadratic programming, and applied explicit MPC to the active suspension system, and the results showed that the suspension performance was significantly better than that of skyhook control suspension. However, the prediction parameters are fixed and cannot adapt to the changing road conditions. Bououden S et al. [17] proposed a new method for the design of robust nonlinear multivariable predictive control of nonlinear active suspension with the TS fuzzy method. By transforming the controller design into a convex optimization problem with linear matrix inequality constraints, the robustness of the suspension control system has been improved. However, it has the disadvantage of too much computation. At present, the application of MPC on vehicle suspension control mainly focuses on the implementation of the explicit solution of MPC and road identification, and the parameters of the designed MPC method are fixed and unchanged, it can only realize the optimal control effect of local specific road conditions, but not the optimal global road conditions. Therefore, it is necessary to study the relationship between the MPC parameters and the suspension system’s damping control effect.

Air springs have the advantages of low natural vibration frequency, adjustable stiffness, and lightweight, and they are increasingly widely used in engineering [18]. Air suspensions are categorized into passive air suspensions, active air suspensions, and semi-active air suspensions, depending on whether they require energy input or not [19]. Many scholars in China have chosen to add additional air chambers to provide air sources for semi-active suspension with air springs, which can achieve the effect of changing the stiffness of air suspension [20,21]. The variable stiffness air springs can be adjusted to optimize the stiffness and damping of the suspension, suppressing attitude changes and improving handling stability and ride comfort [22]. Air springs have more advantages than coil springs. Air springs have ideal inverse “S” stiffness characteristics, low natural frequency, and superior vibration isolation performance. The combination of passive air springs and magnetorheological semi-active suspension means that the suspension has a lower stiffness and controllable damping, which can better improve the vibration isolation performance of the vehicle suspension system.

In this paper, the magnetorheological damper is utilized as the damping component in the semi-active air suspension system, while a passive air spring is employed in conjunction with the magnetorheological semi-active suspension with the air spring system. By reducing the suspension stiffness of the magnetorheological semi-active suspension with an air spring system, it enhances ride comfort. The magnetorheological semi-active suspension with air springs is taken as the research object to study the semi-active suspension of 1/4 vehicle, and the two-degree-of-freedom model of 1/4 vehicle means that it retains some of the essential features of the suspension and avoids the complexity of the research on the model of the whole vehicle [23]. The mechanical characteristics of the magnetorheological damper of the research group are experimented with by building the experimental rig of the magnetorheological damper. The forward model of the magnetorheological damper is established based on the improved hyperbolic tangent model, and the inverse model of the magnetorheological damper is established using the adaptive fuzzy neural network method. The MPC strategy of magnetorheological semi-active suspension with air springs is designed, and the suspension performance indexes of passive suspension, model predictive control suspension, and variable horizon model predictive control (VHMPC) suspension are compared, analyzed, and studied under the mixed working conditions of random road surface and continuous speed bumps. The existing MPC research mainly focuses on the implementation of explicit solutions of MPC and road recognition, with less research on improving the MPC algorithm itself. Therefore, it is necessary to study more suitable MPC algorithms for suspension applications in terms of model prediction parameters. To solve the problem of the poor control effect of the semi-active suspension model predictive control in complex road conditions, this paper analyzes the characteristics of the horizon and MPC and selects an optimized horizon for different road conditions. It proposes a variable horizon model predictive control (VHMPC) algorithm that automatically switches between horizons based on the frequency of the road input. The implementation of VHMPC effectively enhances the ride comfort of suspension systems under challenging road conditions, thereby presenting a novel approach for integrating MPC into suspension system applications. The specific contributions of this paper are as follows:

(1) The utilization of an adaptive fuzzy neural network in establishing the inverse model of magnetorheological damper, enhances the accuracy of the mathematical representation for its mechanical properties.
(2) Investigation into the relationship between suspension performance and MPC parameters enables optimization of the control effect by selecting optimal horizon parameters for MPC.
(3) Considering different input frequencies from road surfaces, a VHMPC algorithm is proposed to improve suspension performance under complex working conditions.
(4) The proposed VHMPC algorithm enhances ride comfort in semi-active suspension systems operating under complex road conditions.

## 2. Modeling of Magnetorheological Damper

**Figure 1:**
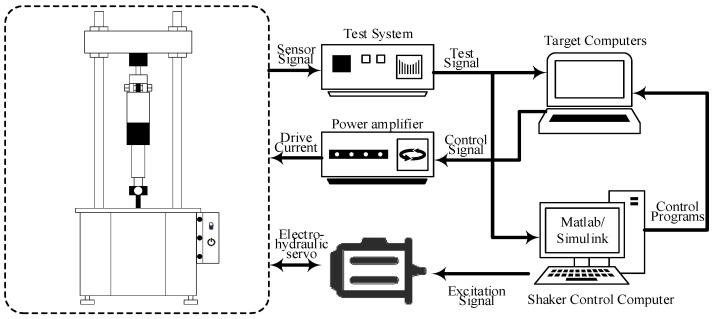
Mechanical performance test system of magnetorheological damper.

**Figure 2:**
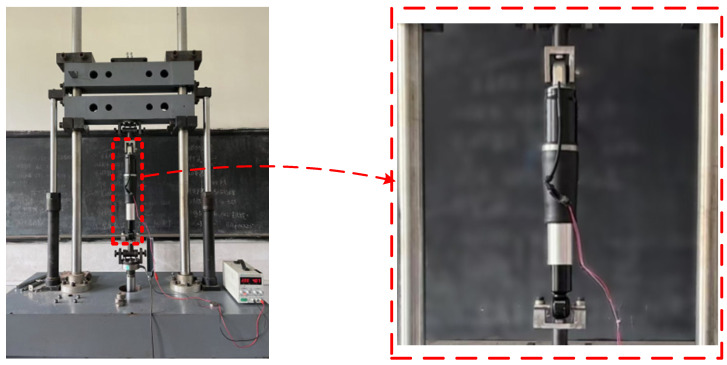
Prototype of mechanical test system and magnetorheological damper.

In order to obtain data on the demonstrative power characteristics of the magnetorheological damper, the demonstrative work characteristics of the magnetorheological damper designed by the research group are tested. The mechanical property test system of the damper shown in Figure 1 is built. It consists of a computer, excitation platform, electro-hydraulic servo system, test system, power amplifier, and magnetorheological damper. The prototype of the mechanical test system and magnetorheological damper is shown in Figure 2. During the test, the mounting fixture on the excitation platform fixes the outer rod of the damper piston and the bottom end of the bar so that the damper bar can do simple harmonic motion with a certain amplitude and frequency relative to the piston. At the same time, a certain level of DC is passed into the damper coil. The combination of different values of simple harmonic displacement excitation amplitude, displacement, and input current completes the dynamic performance test of the magnetorheological damper under different working conditions.

### 2.1. Output Damping Force Versus Current Test

**Figure 3:**
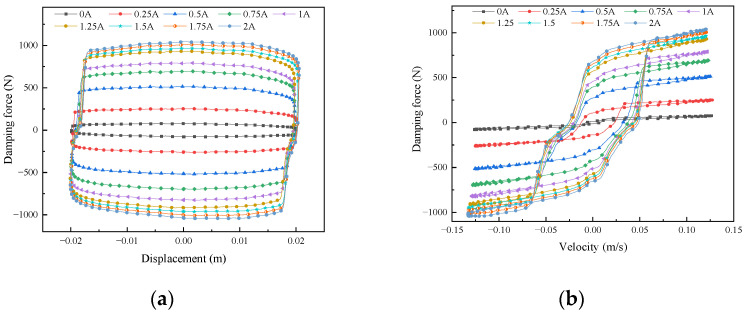
Mechanical characteristics of magnetorheological damper under different currents. (**a**) Damping force-displacement. (**b**) Damping force-velocity.

The amplitude and frequency of the sinusoidal excitation of the excitation platform are unchanged, the amplitude is set to 20 mm, the frequency is set to 1 Hz, and the input current is fed to the magnetorheological damper in the magnitudes of 0, 0.25, 0.5, 0.75, 1, 1.25, 1.5, 1.75, and 2 A. The change in the magnetorheological damper’s output damping force is tested when the excitation current’s value is varied. The mechanical characteristic curves of the damping force versus piston displacement and velocity were obtained, as shown in Figure 3.

As can be seen from Figure 3, the force-displacement curve of the damper exhibits an elliptical shape, the damping force increases with the increase of current, and the hysteresis curve is full with strong energy dissipation ability; the damping force-velocity curve has strong hysteresis characteristics, and the damping force increases slightly with the increase in speed; When the current is 1.5 A, the output damping force of the damper reaches 1000 N, which shows that the damper can meet the application requirements of vehicle suspension.

### 2.2. Dynamic Performance Test

**Figure 4:**
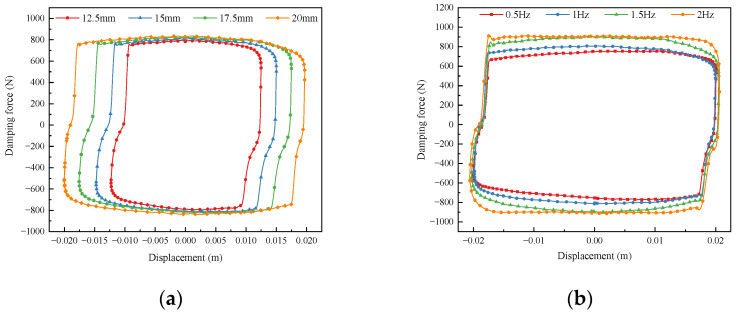
The damping force under different frequencies and amplitudes. (**a**) Damping force-diplacement at different amplitudes. (**b**) Damping force-displacement at different frequencies.

In order to investigate the influence of the amplitude and frequency of the input excitation on the output damping force of the damper, the sinusoidal excitation frequency of the test platform was set to 1 Hz. The input current of the magnetorheological damper was set to 1 A. The amplitude of the excitation was varied in increments of 2.5 mm from 12.5 mm to 20 mm, which resulted in the relationship between the output force of the damper and the size of the amplitude, as shown in Figure 4a. The sinusoidal excitation amplitude of the test platform was set to 20 mm, the magnetorheological damper input current was set to 1 A, and the excitation frequency was varied in 0.5 Hz increments from 0.5 Hz to 2 Hz. The relationship between the output force of the damper and the magnitude of the frequency was measured, as shown in Figure 4b.

From Figure 4, it can be seen that the output damping force increases slightly with the increase of excitation frequency, and the output damping force increases slightly with the increase of excitation amplitude. This is due to the limited conditions. The increase in the excitation frequency and amplitude will lead to an increase in the internal motion velocity of the damper, increasing the viscous damping force and resulting in a slight increase in the output damping force of the damper.

From the above analysis, the magnetorheological damper in the applied excitation has the greatest influence on the output damping force, while the frequency and amplitude have less influence. Therefore, in the subsequent damper modeling and suspension control research, the input current of the damper is used as the control quantity to achieve real-time control of the magnetorheological damper.

### 2.3. The Forward Modeling of Magnetorheological Dampers

Due to the viscous property and magnetorheological effect of the magnetorheological fluid in the magnetorheological damper, the mechanical properties of the magnetorheological damper are related to the magnetic field strength, damper speed, displacement amplitude, frequency, etc. Therefore, the magnetorheological damper has strong nonlinear and hysteresis characteristics, and its mathematical model’s accuracy significantly influences the control effect of the magnetorheological damper.

The hyperbolic tangent model effectively describes the hysteresis characteristics of MRD while maintaining a simple structure that is easy to use and identify due to its fewer parameters. Consequently, it finds wide application in semi-active control systems.

In comparison to the original hyperbolic tangent model, the improved version comprehensively considers both damping characteristics at high speeds and nonlinear/hysteretic behaviors at low speeds. Additionally, this enhanced model reduces the parameter count to five, facilitating easier parameter identification. The improved hyperbolic tangent model consists of elastic, damping elements and a hyperbolic tangent model connected in series and parallel, which can describe the characteristics of the magnetorheological damper before and after yielding. The improved hyperbolic tangent model requires fewer parameters to be identified, and the physical meaning is clear, so this paper uses the improved hyperbolic tangent model as the mathematical expression model of the magnetorheological damper with the following expressions:

(1) $$F=a_{1}\tanh(a_{2}(\dot{x}+kx))+a_{3}(\dot{x}+kx)+f_{0}$$

where: $a_{1}$, k, is the scale factor, in which $a_{1}$ is the hyperbolic tangent coefficient related to the current size; k is the elastic modulus coefficient, the value of which affects the hysteresis loop width; $a_{2}$, $a_{3}$ is the damping coefficient, respectively, affecting the curve trend of the pre-yield area and the post-yield area; $f_{0}$ is the bias damping force.

The improved hyperbolic tangent mathematical model needs to identify a total of five parameters: $a_{1}$, $a_{2}$, k, $a_{3}$, and $f_{0}$. From the above analysis of the dynamic characteristics of the magnetorheological damper, it can be seen that the input current has the most significant influence on the damping force of the magnetorheological damper, which is much larger than the influence of other excitation factors on the damping force, so it is aimed at controlling the control current to control the magnetorheological damper. In this paper, based on the particle swarm algorithm, the improved hyperbolic tangent model parameters are parameterized by the experimental data of the magnetorheological damper with five groups of control currents from 0 to 1 A, respectively.

The identification principle states that the enhanced hyperbolic tangential model can accurately depict the mechanical properties of MRD. Therefore, the objective of identification is to minimize the discrepancy between the experimental data of damping force for each current group and the predictive force from the mathematical model. Consequently, we select the fitness function for particle swarm as follows:

(2) $$f=\sum_{i=0}^{m}{\left(F_{i}-{\hat{F}}_{i}\right)}^{2}$$

The damping force of the *i*-th test data is represented by *F_i_*, while the damping force calculated by the MRD model under the *i*-th particle parameter vector is represented by *F_i_*. Here, *m* represents the number of test data used for identification.

**Figure 5:**
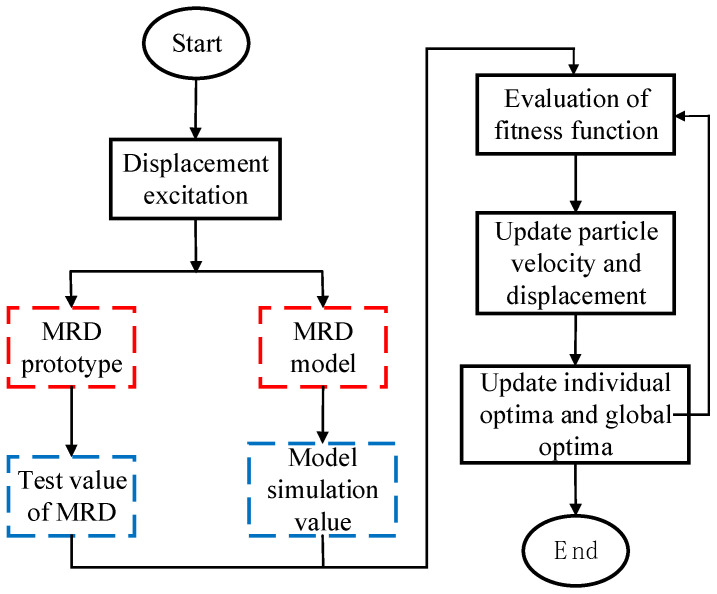
Particle swarm optimization algorithm identification process.

The process of identifying model parameters using particle swarm optimization is illustrated as follows in Figure 5:

The model parameters are identified using the particle swarm optimization algorithm (PSO), inspired by bird flocks’ foraging behavior. They can be considered a flock of particles searching within a search range, with each particle representing a solution vector. Individual optimal and group optimal solutions within the population are iteratively updated during the search, and this information interaction enables the entire group to derive an optimal solution vector. The mathematical expression of particle velocity is

(3) $$v_{i}(t+1)=wv_{i}(t)+c_{1}r_{1}[p_{iBest}-p_{i}(t)]+c_{2}r_{2}[g_{Best}-p_{i}(t)]$$

The variables $p_{i}$ and $p_{iBest}$ represent the current position of the *i*-th particle and its optimal position in terms of individual experience, respectively. Meanwhile, $g_{Best}$ represents the optimal position based on the overall experience of the population. The speed of *i*-th particle is denoted as $v_{i}$, with w representing inertia weight and $r_{1}$ and $r_{2}$ being random numbers within [0, 1]. By considering both the optimal positions of each particle and that of the population as a whole, we obtain a new search speed $v_{i}(t+1)$, the particle position is then updated to

(4) $$p_{i}(t+1)=p_{i}(t)+v_{i}(t+1)$$

The parameters of the particle swarm optimization algorithm are determined as follows: the search range for each parameter to be identified depends on the actual estimation. The inertia weight w influences the exploration ability of the population in the PSO algorithm and determines both the speed at which particles renew their positions and their level of self-awareness. To comprehensively consider both individual particle optimization and overall population optimization, a value of 0.86 is assigned to the inertia weight w. The acceleration factors $c_{1}$ and $c_{2}$ represent the influence of individual particle experience and collective population experience, respectively. The values for $c_{1}$ and $c_{2}$ are set as 2.0 and 1.8, respectively. The number of particles in the swarm is set to 100, with a total of 100 iterations performed.

Based on experimental data from MRD consisting of five groups with control currents ranging from 0 to 1 A, Table 1 shows the identified parameters corresponding to each current value.

By analyzing the data in the table, it can be observed that the parameters $a_{2}$, k, and $f_{0}$ remain relatively unaffected by current and can be regarded as constant variables independent of current. Therefore, by calculating the average values of these parameters for each group at different currents, we obtain $a_{2}=50.37$, k=1.109, and $f_{0}=-7.456$. On the other hand, significant variations are observed in the values of $a_{1}$ and $a_{3}$ with respect to current. By examining the parameter identification results under different currents to investigate their dependence on current, it is evident that both $a_{1}$ and $a_{3}$ exhibit linear correlations with current. After performing linear regression analysis, we establish the following relationships between parameters and current:

(5) $$\left\{\begin{array}{c} a_{1}=574.88I+40.23\\ a_{3}=1469.36I+376.13 \end{array}\right.$$

After identification, five parameters of the improved hyperbolic tangent model can be determined, and the improved hyperbolic tangent mathematical model of the magnetorheological damper after identification is obtained as

(6) $$F=\left(574.88I+40.23\right)tanh(50.37(\dot{x}+kx))+(1469.36I+376.13)(\dot{x}+kx)+f_{0}$$

In order to verify the accuracy of the model in describing the mechanical properties of the magnetorheological damper, based on the mathematical model identified in the above equation, the same current value, velocity, and displacement as the experiment are inputted into it and the damping force of the magnetorheological damper is obtained. The simulation value of the magnetorheological damper is obtained by inputting the same current value, velocity, and displacement as the experiment. The accuracy of the established mathematical model can be verified.

**Figure 6:**
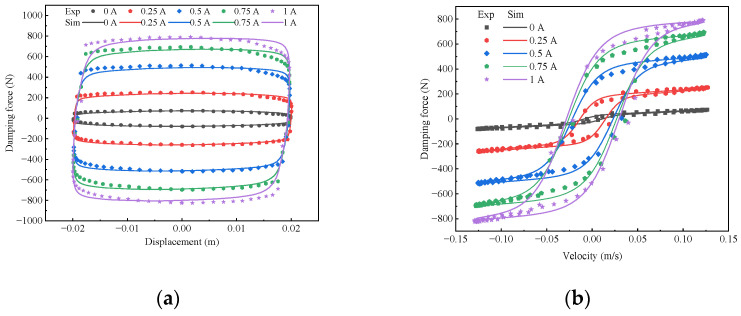
Comparison between the simulation results of the forward model and the experimental data. (**a**) Damping force-displacement. (**b**) Damping force-velocity.

According to the identified improved hyperbolic tangent mathematical model of the magnetorheological damper of Equation (2), the model simulation values of the damping force corresponding to the experimental data are simulated, and the results are shown in Figure 6. From Figure 6, it can be seen that the improved hyperbolic tangent model obtained from the identification can be highly fitted to the experimental data and can well describe the mechanical properties and hysteresis characteristics of the magnetorheological damper. Therefore, the established improved hyperbolic tangent mathematical model of the magnetorheological damper has high accuracy and can be used in the subsequent research of the magnetorheological semi-active suspension with air springs control strategy.

### 2.4. Inverse Model of Magnetorheological Damper

In the practical magnetorheological semi-active suspension system, the controller calculates the desired damping force of the magnetorheological damper according to the state of the vehicle. The input to the magnetorheological damper as an actuator is a current, i.e., the current magnitude is relied upon to control the output damping force of the magnetorheological damper. Therefore, it is necessary to establish an inverse model of the magnetorheological damper between the controller and the magnetorheological damper so that the desired damping force can be converted into the input current. The current that needs to be applied to the magnetorheological damper is deduced from the operating state of the vehicle suspension system and the desired damping force required to obtain the accurate actual output damping force of the magnetorheological damper.

Given the strong nonlinearity of the magnetorheological damper, this paper utilizes an adaptive neuro-fuzzy inference system (ANFIS) to establish an inverse model of the magnetorheological damper. ANFIS is a kind of neural network and fuzzy algorithm fusion of a kind of intelligent algorithm, combining the characteristics of the neural network adaptive self-learning and fuzzy algorithms without the need for a priori model without relying on the precise mathematical model, suitable for nonlinear systems and complex systems of nonparametric modeling.

**Figure 7:**
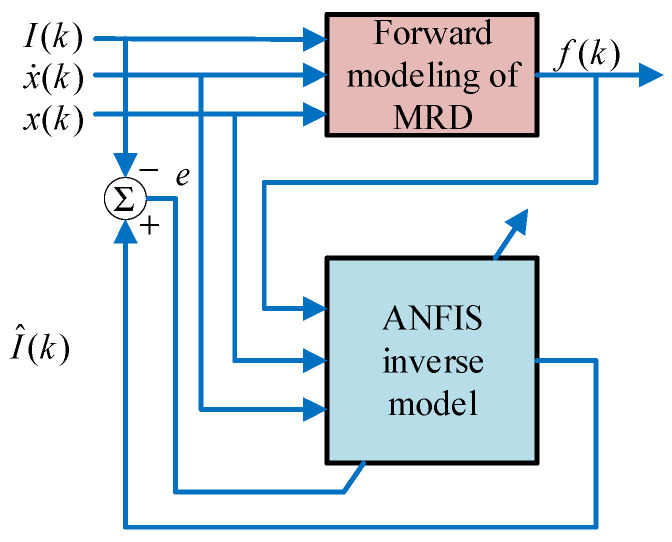
ANFIS inverse model of magnetorheological damper.

The displacement, velocity, and desired damping force data of the magnetorheological damper piston are used as inputs by the inverse model system to determine the input current of the magnetorheological damper. The principle of the adaptive neuro-fuzzy inference inverse model of magnetorheological damper established in this paper is shown in Figure 7. The experimental data of displacement, velocity, and damping force under the specific current of the magnetorheological damper are used as the input of the ANFIS inference system. The output of the inverse model is the inference current. The inference system is then corrected continuously according to the error of the inference current and the experimental current until the inference system achieves a certain level of accuracy.

The experimental data from the magnetorheological damper were divided into two large groups in chronological order, each of which contained experimental data from five groups within a current of 0 to 1 A; The first large group is used as the training data to establish the ANFIS inverse inference model of the magnetorheological damper, and the other large group is used as the test data to check the accuracy of the inverse model.

**Figure 8:**
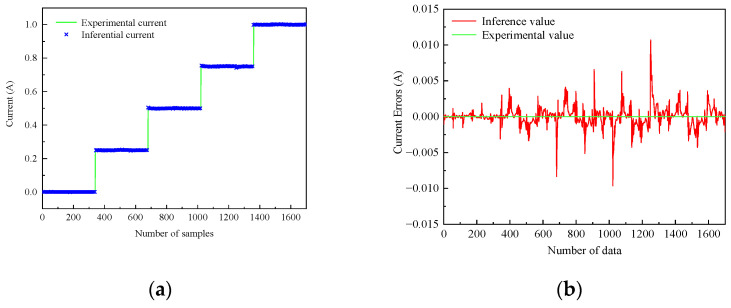
Training data validation of ANFIS inverse model (**a**) Tracking data. (**b**) Tracking error.

Figure 8 shows the comparison between the experimental values and the inference values in the training data of the inverse model of the magnetorheological damper. It can be seen that the ANFIS inverse model of the magnetorheological damper in the training data can track the experimental values very well, and the inference error is within 0.01 A, which is an excellent inference accuracy.

**Figure 9:**
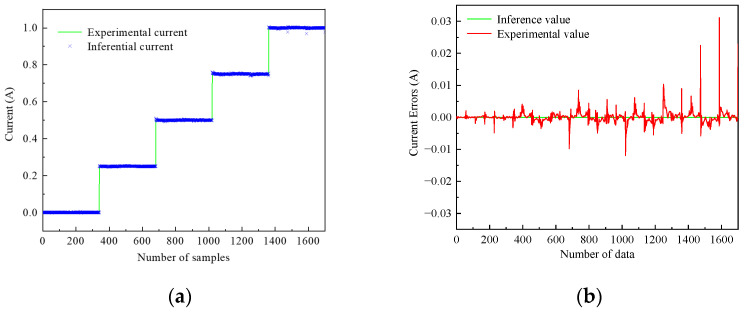
Checking data validation of ANFIS inverse model. (**a**) Checking data. (**b**) Checking error.

Figure 9 shows the comparison between the experimental values and the inference values in the checking data of the inverse model of the magnetorheological damper. As can be seen from Figure 9, the inference error of the test data is larger than that of the training data, but it is still able to track the experimental value well. The inference error is within 0.03 A, which has good inference accuracy. Therefore, the established ANFIS inverse model of the magnetorheological damper can be applied in the VHMPC of magnetorheological semi-active suspension with air springs.

## 3. Modeling of Semi-Active Air Suspension

### 3.1. Modeling of Air Spring

The ideal inverse “S” curve stiffness characteristics of air springs are due to the nonlinear properties of the structure and materials. The inverse “S” curve characteristic means that when the air spring works near the static load, the air spring shows more negligible stiffness and minor suspension bias frequency. When the suspension is excited by the road surface more vigorously, the air spring in the suspension is compressed and pulled by a larger amplitude; the air spring shows greater stiffness and more energy from the road surface can be absorbed under the limited suspension travel of the suspension.

**Figure 10:**
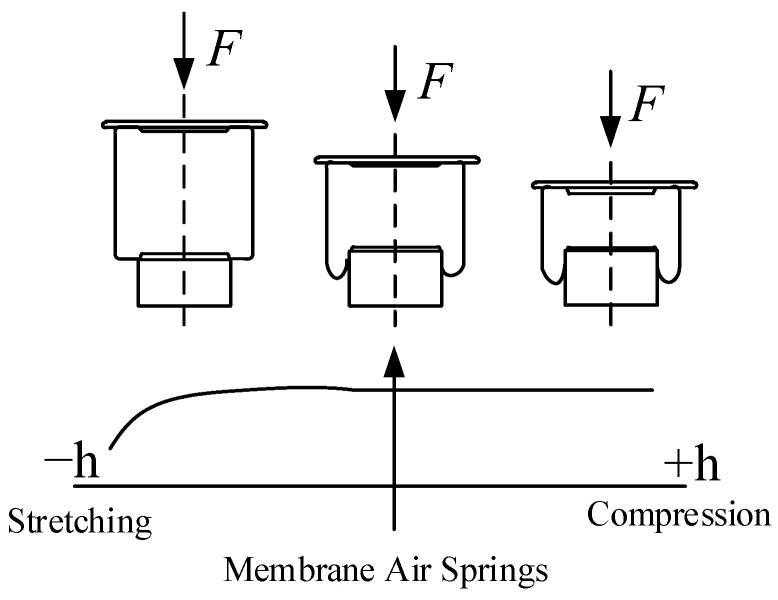
Working principle of air spring.

Compared with coil springs, air springs have the advantages of low natural vibration frequency, adjustable stiffness, long service life, and low noise. In addition, air springs have nonlinear muscular stiffness characteristics, and they can show better wheel grip when driving in complex road conditions. When the suspension spring mass changes, its intrinsic frequency does not change much [24]. The schematic structure of the air spring is shown in Figure 10.

The air spring consists of a cover plate, a rubber airbag, and a piston base, which forms a confined space. The pressure inside the air spring is set as *p*, the atmospheric pressure is set as *p_a_*, and the effective area is set as *A_e_*. Then, the output force of the gas spring is:

(7) $$F=\left(p-p_{a}\right)A_{e}$$

From Hooke’s law, the relationship between the stiffness *k* of the air spring and the variation of the spring height *h* is derived:

(8) $$k=\frac{dF}{dh}=\frac{dp}{dh}A_{e}+(p-p_{a})\frac{dA_{e}}{ds}$$

In the process of working the air spring, the deformation speed of the rubber air bladder of the spring is less than a few meters per second, which is much lower than the speed of transmission of pressure waves of the air inside the air bladder, and much lower than the speed of movement of the air molecules inside the air bladder, which are all in the order of hundreds of meters per second. Therefore, the localized imbalance of the air inside the spring capsule that occurs by the air spring during its operation can be eliminated very quickly, and all the air inside the air spring can be considered to be in the same thermodynamic state.

The state of the air in the airbag conforms to the equation of state for an ideal air,

(9) $$pV^{m}=K$$

where the equation *K* is a constant, *V* is the volume of the air spring, and *m* is the air multiplicity index.

Deriving both ends of Equation (9) with respect to the height of the spring. It can be obtained as:

(10) $$\frac{dp}{dh}=-\frac{pm}{V}\frac{dV}{dh}$$

Based on the properties of the gas pocket in the gas spring

(11) $$\frac{dV}{dh}=A_{e}$$

Substituting Equations (10) and (11) into Equation (8) yields.

(12) $$k=-\frac{pm}{V}A_{e}^{2}+(p-p_{a})\frac{dA_{e}}{dh}$$

For airbags in air suspension, there is

(13) $$\frac{dA_{e}}{dh}\approx 0$$

From this, the stiffness of the air spring is obtained as

(14) $$k=-\frac{pm}{V}A_{e}^{2}$$

Equation (14) shows that the stiffness of the air spring is related to the structural parameters of its effective pressure and air bag volume. The characteristic parameters of the air spring used in this paper are shown in Table 2.

### 3.2. Modeling of Magnetorheological Semi-Active Air Suspension

**Figure 11:**
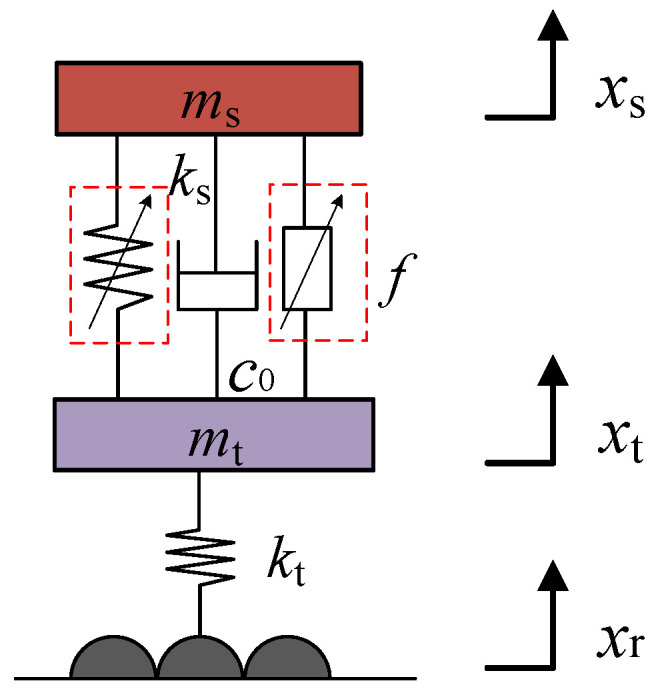
Model of magnetorheological semi-active air suspension for 1/4 vehicle.

Suspension vertical motion and road surface input vibration are the main factors affecting the suspension system in the design and development stage of the damper, usually using a 1/4 vehicle two-degree-of-freedom suspension system for computer simulation analysis and physical bench testing. In order to verify the rationality and feasibility of the designed suspension damper. This paper focuses on the 1/4 vehicle two-degree-of-freedom magnetorheological semi-active suspension with air springs, whose simplified model is shown in Figure 11. In Figure 11, *m_s_*: the mass of the vehicle body; *m_t_*: the mass of the wheels; *x_s_*: the absolute displacement of the vehicle body; *x_t_*: the absolute displacement of the wheels; *x_r_*: the road excitation; *k_s_*: the equivalent stiffness of the air compression springs; *k_t_*: the equivalent stiffness of the wheels; *c*_0_: the friction damping coefficient; *f*: the output of the magnetorheological damper to control the damping force. In Figure 11, the air spring and magnetorheological damper are arranged in parallel on the suspension system. The mathematical model of the air spring is established, and then the linkage simulation of the suspension system is realized with the magnetorheological damper in the form of simulation object.

From the mechanical model established in Figure 11, the differential equation of motion of the suspension system can be established according to Newton’s dynamics law:

(15) $$\left\{\begin{array}{c} m_{s}{\ddot{x}}_{s}+c_{0}\left({\dot{x}}_{s}-{\dot{x}}_{t}\right)+k_{s}\left(x_{s}-x_{t}\right)+f=0\;\\ m_{t}{\ddot{x}}_{t}-c_{0}\left({\dot{x}}_{s}-{\dot{x}}_{t}\right)-k_{s}\left(x_{s}-x_{t}\right)+k_{t}\left(x_{t}-x_{r}\right)-f=0 \end{array}\right.$$

where: ${\ddot{x}}_{s}$ is the vertical acceleration of the body; ${\ddot{x}}_{t}$ is the wheel’s vertical acceleration; ${\dot{x}}_{s}$ is the vertical velocity of the body; ${\dot{x}}_{t}$ is the wheel vertical velocity.

To facilitate the design of the controller, the state vector of the magnetorheological semi-active air suspension with air spring is selected as for:

(16) $$X={\left[\begin{array}{cccc} x_{s} & x_{t} & {\dot{x}}_{s} & {\dot{x}}_{t} \end{array}\right]}^{T}$$

The differential equations of motion of the magnetorheological semi-active suspension with air springs are transformed into the system state equation and output equation:

(17) $$\dot{X}=\tilde{A}X+\tilde{B}U+\tilde{\Gamma }W$$

(18) $$Y=\tilde{C}X+\tilde{D}U+\tilde{\Theta }W$$

In the above equation: $\tilde{A}=\left[\begin{array}{cccc} 0 & 0 & 1 & 0\\ 0 & 0 & 0 & 1\\ -\frac{k_{s}}{m_{s}} & \frac{k_{s}}{m_{s}} & -\frac{c_{0}}{m_{s}} & \frac{c_{0}}{m_{s}}\\ \frac{k_{s}}{m_{t}} & -\frac{k_{s}+k_{t}}{m_{t}} & \frac{c_{0}}{m_{t}} & -\frac{c_{0}}{m_{t}} \end{array}\right]$; $\tilde{B}=\left[\begin{array}{c} 0\\ 0\\ -\frac{1}{m_{s}}\\ \frac{1}{m_{t}} \end{array}\right]$; $U=\left[f\right]$;

$\tilde{\Gamma }=\left[\begin{array}{c} 0\\ 0\\ 0\\ \frac{k_{t}}{m_{t}} \end{array}\right]$; $W=\left[x_{r}\right]$; $Y=\left[\begin{array}{c} {\ddot{x}}_{s}\\ x_{t}-x_{r}\\ x_{s}-x_{t} \end{array}\right]$; $\tilde{C}=\left[\begin{array}{cccc} -\frac{k_{s}}{m_{s}} & \frac{k_{s}}{m_{s}} & -\frac{c_{0}}{m_{s}} & \frac{c_{0}}{m_{s}}\\ 0 & 1 & 0 & 0\\ 1 & -1 & 0 & 0 \end{array}\right]$; $\tilde{D}=\left[\begin{array}{c} -\frac{1}{m_{s}}\\ 0\\ 0 \end{array}\right]$; $\tilde{\Theta }=\left[\begin{array}{c} 0\\ -1\\ 0 \end{array}\right]$.

## 4. Design of Variable Horizon Model Predictive Controller

MPC is an advanced control strategy widely used in the field of industrial control, and its unique feature is the use of optimization methods to solve the control problem. Traditional optimal control algorithms calculate the optimal control quantity based on the infinite horizon during the control process and cannot deal with the outputs, states, and output constraints appearing in the process, so they cannot achieve the optimal control effect. The basic idea of MPC is to use the state of the system at the current moment, the input variables for prediction, and solve the finite horizon optimal control problem to obtain a set of optimal control input sequences. Subsequently, the first set of results in the optimal control sequence is extracted and applied to the controlled object. At the next sampling moment, the same process is repeated, and the measured state information is used as the initial state at the next sampling moment to predict the future dynamics of the system, and the control quantities at the next moment are obtained by solving the optimal control problem again. VHMPC is a control algorithm based on MPC that can adaptively switch between the prediction horizon and control horizon.

### 4.1. Design of Model Predictive Controller

According to the MPC theory, the continuous system is discretized using the zero-order hold discretization method [25].

(19) $$\begin{array}{c} x\left(k+1\right)=A_{d}x\left(k\right)+B_{d}u\left(k\right)+\Gamma_{d}w\left(k\right)\\ y\left(k\right)=C_{d}x\left(k\right)+D_{d}u\left(k\right)+\Theta_{d}w\left(k\right) \end{array}$$

where $A_{d}=(I+T\tilde{A})$, $B=T\tilde{B}$, $\Gamma =T\tilde{\Gamma }$, $C=\tilde{C}$, $D=\tilde{D}$, T is the sampling period.

Let the prediction horizon be $N_{p}$, and the control horizon be $N_{c}$. Firstly, define the following matrix:

(20) $$X\left(k\right)={\left[x\left(k+1|k\right),x\left(k+2|k\right),\cdots ,x(k+N_{p}|k)\right]}^{T}$$

(21) $$Y\left(k\right)={\left[y\left(k+1|k\right),y\left(k+2|k\right),\cdots ,y(k+N_{p}|k)\right]}^{T}$$

(22) $$U\left(k\right)={\left[u\left(k|k\right),u\left(k+1|k\right),\cdots u(k+N_{c}|k)\right]}^{T}$$

(23) $$W\left(k\right)=[w\left(k|k\right),w\left(K+1|k\right),\cdots ,w(k+N_{p}-1|k)]$$

In the above equation, $x\left(k\right)$ represents the actual state of the system at moment k, x(k+i|k) represents the system’s predicted value of the system state at moment k for moment k+i, u(k+i|k) represents the system’s predicted amount of system control at moment k for moment k+i, and y(k+i|k) represents the system’s predicted output of the system for moment k for moment k+i.

The state variables of the discrete system can be obtained from the state equations:

(24) $$\begin{array}{l} \;\;\;x\left(k+1|k\right)=A_{d}x\left(k\right)+B_{d}u\left(k|k\right)+\Gamma_{d}w\left(k\right)\\ \;\;x\left(k+2|k\right)=A_{d}^{2}x\left(k\right)+A_{d}B_{d}u\left(k|k\right)+B_{d}u\left(k+1|k\right)+A_{d}\Gamma_{d}w\left(k|k\right)+\Gamma_{d}w(k+1|k)\\ \vdots \\ x\left(k+N_{p}|k\right)=A_{d}^{N_{p}}x\left(k\right)+A^{N_{p}-1}A_{d}^{N_{p}-1}B_{d}u\left(k|k\right)+A_{d}^{N_{p}-2}B_{d}u\left(k+1|k\right)+\cdots \\ \;\;\;\;\;\;\;+\sum_{i=1}^{N_{p}-N_{c}}A_{d}^{i-1}B_{d}u\left(k+N_{c}|k\right)+A_{d}^{N_{p}-1}\Gamma_{d}w(k|k)+A_{d}^{N_{p}-2}\Gamma_{d}w\left(k+1|k\right)\\ \;\;\;\;\;\;\;+\cdots +\Gamma_{d}w\left(k+N_{p}-1|k\right) \end{array}$$

The output variables are derived from the state equation:

(25) $$\begin{array}{l} \;y\left(k+1|k\right)=C_{d}A_{d}x\left(k\right)+C_{d}B_{d}u\left(k|k\right)+D_{d}u\left(k+1|k\right)+C_{d}\Gamma_{d}w\left(k\right)\\ \;\;\;y\left(k+2|k\right)=C_{d}A_{d}^{2}x\left(k\right)+C_{d}A_{d}B_{d}u\left(k|k\right)+C_{d}B_{d}u\left(k+1|k\right)+D_{d}u\left(k+2|k\right)\\ \;\;\;\;\;\;\;+C_{d}A_{d}\Gamma_{d}w\left(k|k\right)+C_{d}\Gamma_{d}w(k+1|k)\\ \vdots \\ y\left(k+N_{p}|k\right)=C_{d}A_{d}^{N_{p}}x\left(k\right)+C_{d}A_{d}^{N_{p}-1}B_{d}u\left(k|k\right)+C_{d}A_{d}^{N_{p}-2}B_{d}u\left(k+1|k\right)+\cdots \\ \;\;\;\;\;\;+\sum_{i=1}^{N_{p}-N_{c}}C_{d}A_{d}^{i-1}B_{d}u\left(k+N_{c}|k\right)+D_{d}u\left(k+N_{c}|k\right)+C_{d}A_{d}^{N_{p}-1}\Gamma_{d}w(k|k)\\ \;\;\;\;\;\;+C_{d}A_{d}^{N_{p}-2}\Gamma_{d}w(k+1|k)+\cdots C_{d}\Gamma_{d}w\left(k+N_{p}-1|k\right) \end{array}$$

The state prediction and output prediction at the moment *k* are written as the following expression:

(26) $$\begin{array}{c} X\left(k\right)=\bar{A}x\left(k\right)+\bar{B}U\left(k\right)+\bar{\Gamma }W(k)\\ Y\left(k\right)=\bar{C}x\left(k\right)+\bar{D}U\left(k\right)+\bar{\Theta }W(k) \end{array}$$

where: $\bar{A}={\left[\begin{array}{c} A_{d}\\ A_{d}^{2}\\ \vdots \\ A_{d}^{N_{p}} \end{array}\right]}_{(N_{p})\times 1};\;\bar{B}={\left[\begin{array}{cccc} B_{d} & 0 & \cdots & 0\\ A_{d}B_{d} & B_{d} & \cdots & 0\\ \vdots & \vdots & \ddots & \vdots \\ A_{d}^{N_{p}-1}B_{d} & A_{d}^{N_{p-2}}B_{d} & \cdots & \sum_{i=1}^{N_{p}-N_{c}}A_{d}^{i-1}B_{d} \end{array}\right]}_{N_{p}\times (N_{c}+1)}$;

$\bar{\Gamma }={\left[\begin{array}{cccc} \Gamma_{d} & 0 & \cdots & 0\\ A_{d}\Gamma_{d} & \Gamma_{d} & \cdots & 0\\ \vdots & \vdots & \ddots & \vdots \\ A_{d}^{N_{p}-1}\Gamma_{d} & A_{d}^{N_{p}-2}\Gamma_{d} & \cdots & \Gamma_{d} \end{array}\right]}_{N_{p}\times N_{p}};\;\bar{C}={\left[\begin{array}{c} C_{d}A_{d}\\ C_{d}A_{d}^{2}\\ \vdots \\ C_{d}A_{d}^{N_{p}} \end{array}\right]}_{N_{p}\times 1}$;

$\bar{D}={\left[\begin{array}{cccc} C_{d}B_{d} & D_{d} & \cdots & 0\\ C_{d}A_{d}B_{d} & C_{d}B_{d} & \cdots & 0\\ \vdots & \vdots & \ddots & \vdots \\ C_{d}A_{d}^{N_{p}-1}B_{d} & C_{d}A_{d}^{N_{p}-2}B_{d} & \cdots & D_{d}+\sum_{i=1}^{N_{p}-N_{c}}C_{d}A_{d}^{i-1}B_{d} \end{array}\right]}_{N_{p}\times (N_{c}+1)}$;

$\bar{\Theta }={\left[\begin{array}{cccc} C_{d}\Gamma_{d} & 0 & \cdots & 0\\ C_{d}A_{d}\Gamma_{d} & C_{d}\Gamma_{d} & \cdots & 0\\ \vdots & \vdots & \ddots & \vdots \\ C_{d}A_{d}^{N_{p}-1}\Gamma_{d} & C_{d}A_{d}^{N_{p}-2}\Gamma_{d} & \cdots & C_{d}\Gamma_{d} \end{array}\right]}_{N_{p}\times N_{p}}$.

The output of the system is taken as the control object, and also, to make the control output as small as possible, the objective function is established as shown below with the following expression:

(27) $$J={\left(Y\left(k\right)-Y_{ref}\left(k\right)\right)}^{T}Q\left(Y\left(x\right)-Y_{ref}\left(k\right)\right)+{U\left(k\right)}^{T}RU\left(k\right)$$

where: $Y_{ref}(k)$ represents the reference output, Q represents the weight matrix of the operational cost, and R represents the weight matrix of the control volume cost.

Let $x_{ref}(k)$ be the reference state at moment k, from which the deviation $\bar{E}(k)$ is defined as:

(28) $$\bar{E}\left(k\right)=\bar{C}x\left(k\right)-\bar{C}x_{ref}(k)$$

The reference state output of the system at this time is:

(29) $$Y_{ref}\left(k\right)=\bar{C}x_{ref}\left(k\right)+\bar{\Theta }W(k)$$

The deviation of the system control output from the reference output is then derived as:

(30) $$Y\left(k\right)-Y_{ref}\left(k\right)=\bar{E}\left(k\right)+\bar{D}U(k)$$

The deviation obtained from the above equation is substituted into the objective function to obtain:

(31) $$J={\left(\bar{E}\left(k\right)+\bar{D}U\left(k\right)\right)}^{T}Q\left(\bar{E}\left(K\right)+\bar{D}U\left(k\right)\right)+{U\left(k\right)}^{T}RU\left(k\right)\;={\bar{E}\left(k\right)}^{T}Q\bar{E}\left(k\right)+{U\left(k\right)}^{T}{\bar{D}}^{T}Q\bar{D}U\left(k\right)+2{\bar{E}\left(k\right)}^{T}Q\bar{D}U\left(k\right)+{U\left(k\right)}^{T}RU(k)$$

In the above equation, the term that does not contain *U*(*k*) is an invariant constant when proceeding to solve for the optimal control force, and its removal yields the desired objective function as follows:

(32) $$J={U\left(k\right)}^{T}\left({\bar{D}}^{T}Q\bar{D}+R\right)U\left(k\right)+2{\bar{E}\left(k\right)}^{T}Q\bar{D}U\left(k\right)\;={U\left(k\right)}^{T}\left({\bar{D}}^{T}Q\bar{D}+R\right)U\left(k\right)+2{\left[\bar{C}x\left(k\right)+\bar{\Theta }W\left(k\right)-Y_{ref}\left(k\right)\right]}^{T}Q\bar{D}U\left(k\right)$$

The output variables of the suspension system are constrained with upper and lower limits:

(33) $$L_{y}\leq Y(k)\leq U_{y}$$

i.e.,

(34) $$L_{y}-\bar{D}U\left(k\right)-\bar{\Theta }W\left(k\right)\leq \bar{C}x\left(k\right)\leq U_{y}-\bar{D}U\left(k\right)-\bar{\Theta }W(k)$$

i.e.,

(35) $$\left[\begin{array}{c} \bar{D}\\ -\bar{D} \end{array}\right]U\left(k\right)\leq \left[\begin{array}{c} U_{y}\\ -L_{y} \end{array}\right]+\left[\begin{array}{cc} -\bar{C} & -\bar{\Theta }\\ \bar{C} & \bar{\Theta } \end{array}\right]\left[\begin{array}{c} x(k)\\ W(k) \end{array}\right]$$

The magnetorheological damper output damping force has a certain maximum value, so the controller system should be constrained with upper and lower limits on the input variables.

(36) $$L_{u}\leq U(k)\leq U_{u}$$

After the two constraints are introduced, they are combined with the aforementioned objective function to form a quadratic programming problem.

(37) $${min}_{U\left(k\right)}J={U\left(k\right)}^{T}\left(\bar{D}Q\bar{D}+R\right)U\left(k\right)+2{\left[\bar{C}x\left(k\right)+\bar{\Theta }W\left(k\right)-Y_{ref}\left(k\right)\right]}^{T}Q\bar{D}U\left(k\right)$$

(38) $$s.t.\left[\begin{array}{c} \bar{D}\\ -\bar{D}\\ E\\ -E \end{array}\right]U\left(k\right)\leq \left[\begin{array}{c} U_{y}\\ -L_{y}\\ U_{u}\\ -L_{u} \end{array}\right]+\left[\begin{array}{cc} -\bar{C} & -\bar{\Theta }\\ \bar{C} & \bar{\Theta }\\ 0 & 0\\ 0 & 0 \end{array}\right]\left[\begin{array}{c} x(k)\\ W(k) \end{array}\right]$$

### 4.2. Effect of Prediction Horizon on Suspension Performance

According to the studies of other system models on MPC parameters, the predictive horizon parameters significantly influence the MPC effect. Therefore, the effect of MPC horizon on model predictive control suspension in semi-active air suspension is investigated. Vehicle suspensions primarily work under vertical and high-frequency variable environments. For high-frequency variable road excitation inputs, the traditional MPC is challenging due to the fixed controller parameters to optimize the control effect under variable road conditions. Therefore, the effect of the predictive horizon on the vibration-damping performance of the suspension system is investigated for the predictive horizon of the MPC under different frequency road conditions.

**Figure 12:**
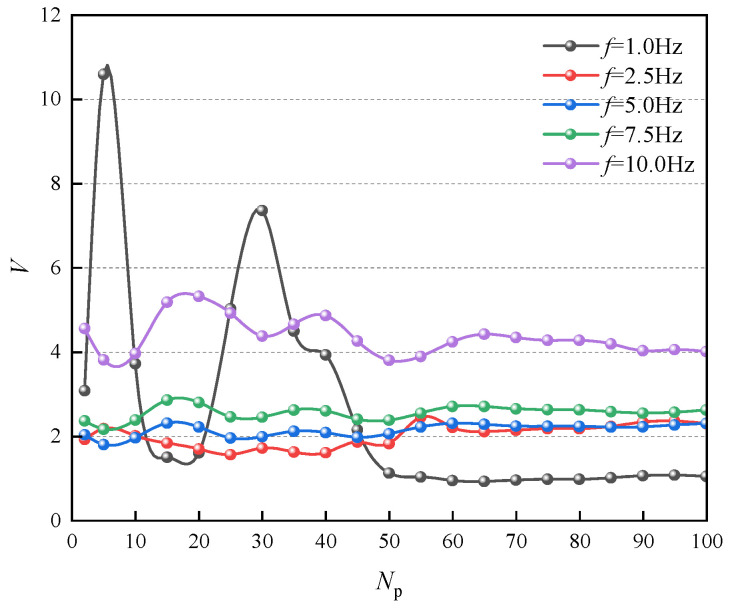
Simulation results under different predictive horizon and road excitation frequencies.

In order to investigate the vibration effects of different vibration frequencies on the MPC suspension, a sinusoidal excitation with an amplitude of 0.05 m and frequencies of 1 Hz, 2.5 Hz, 5 Hz, 7.5 Hz, and 10 Hz are set to simulate the MPC suspension system, and different prediction horizons are set to investigate the relationship between the MPC suspension and the road excitation frequency under different prediction horizons. In order to better evaluate the control effect of the MPC suspension, corresponding evaluation indexes need to be set. The suspension evaluation indexes of the quarter-vehicle suspension model mainly include sprung mass acceleration, dynamic tire deformation, and suspension working space. Therefore, the frequency test evaluation index is set:

(39) $$V=\frac{rms({\ddot{x}}_{s})}{rms({\ddot{x}}_{sp})}+\frac{rms(x_{t}-x_{r})}{rms(x_{tp}-x_{rp})}+\frac{rms(x_{s}-x_{t})}{rms(x_{sp}-x_{tp})}$$

where $rms({\ddot{x}}_{s})$: root mean square (RMS) of sprung mass acceleration, $rms({\ddot{x}}_{sp})$: RMS of sprung mass acceleration of passive suspension, $rms(x_{t}-x_{r})$: RMS of dynamic tire deformation, $rms(x_{tp}-x_{rp})$: RMS of dynamic tire deformation of passive suspension, $rms(x_{s}-x_{t})$: RMS of suspension deflection, $rms(x_{xp}-x_{tp})$: RMS value of suspension deflection of passive suspension. The smaller the value of the evaluation index *V* of the suspension frequency test, the better the comprehensive performance of the suspension and the better the control effect of the controller. The results of different frequencies of sinusoidal excitation input with different predictions of the controller for horizon control simulation are shown in Figure 12.

From Figure 12, it can be seen that different prediction horizons and vibration frequencies have a significant effect on the damping performance of the suspension. As the prediction horizon increases, the vibration-damping effect of the MPC tends to improve with both high-frequency road excitation input and low-frequency road excitation input. The long prediction horizon for low-frequency pavement excitation input can obtain a better damping effect. The short prediction horizon for high-frequency pavement excitation input can obtain a better damping effect.

Therefore, for different frequency pavement excitation conditions, it is necessary to set the parameter horizons in the model prediction controller to variable states in order to achieve better control effects. Therefore, the VHMPC is proposed. The VHMPC can automatically switch the horizon length according to the pavement excitation input frequency level. A frequency sensor measures the required pavement frequency signal.

### 4.3. Optimization of Prediction Horizon Under Different Pavement Conditions

In order to further verify the influence of the prediction horizon on the vehicle suspension performance in actual road conditions, a random road surface model with a vehicle speed of 10 m/s on a class C road surface and a continuous speed bump road surface model with a vehicle speed of 10 m/s are established. They are applied to the road surface excitation inputs of the magnetorheological semi-active air suspension model, respectively. The prediction horizon of the model prediction controller is optimized and analyzed using the suspension vibration evaluation index *V* established above as the measurement basis.

**Figure 13:**
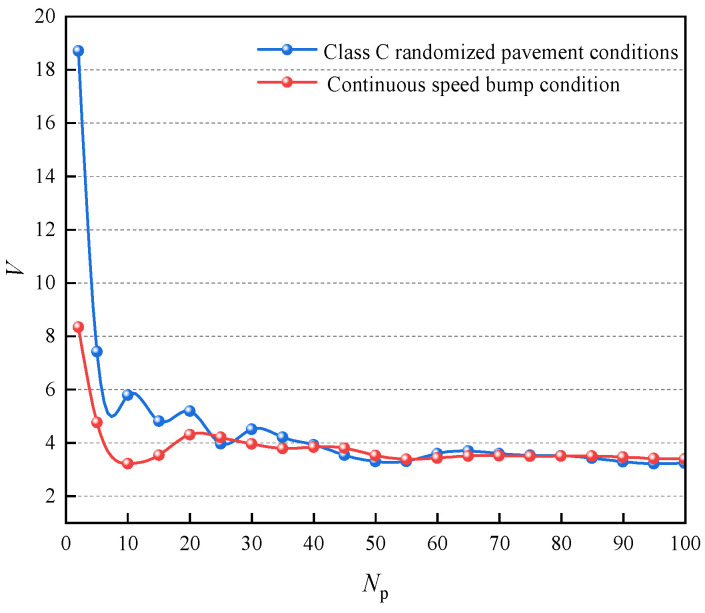
Simulation results under different predictive horizons and input roads.

The suspension vibration damping effect of the magnetorheological semi-active suspension with air springs under continuous speed bump conditions and class C random road conditions is shown in Figure 13. Considering the evaluation index *V* and the computational performance requirements, the optimal value of the prediction horizon for the C-level random road condition is selected as *N_p_*= 55 steps, and the optimal value of the prediction horizon for the continuous deceleration zone condition is selected as *N_p_* = 10 steps.

**Figure 14:**
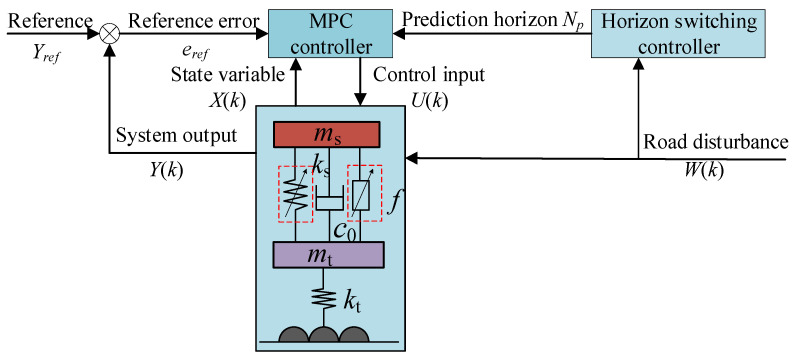
Control block diagram of VHMPC.

As can be seen from Figure 13, the variation trend of the suspension damping effect with the prediction horizon for the C-class random road condition is basically the same as that of the low-frequency sinusoidal excitation with the prediction horizon, and the best part of the damping effect is in the long prediction horizon part. The best prediction horizon in the continuous speed bump condition is *N_p_* = 10 steps; at this time, the C-class random road condition is not the best prediction horizon for the vibration-damping effect. This proves the necessity of VHMPC. The control block diagram of VHMPC is shown in Figure 14.

## 5. Simulation Analysis

The optimal prediction horizon of the MPC suspension is different under different roadway excitation frequencies. Based on this. A VHMPC algorithm that automatically switches the model prediction horizon according to different road conditions is designed. To verify the reasonableness of the designed MPC, the VHMPC is simulated and analyzed.

### 5.1. Mixed Condition

For the magnetorheological semi-active suspension with air springs controlled by the VHMPC, a standard C-class random road surface model and a continuous speed bump road surface model are established. The continuous speed bump model is combined with the standard C-class random road model to reproduce the actual working condition where the vehicle suddenly enters the continuous speed bump while traveling from the random road. The VHMPC is in the long prediction horizon when the vehicle is traveling on the standard C-class random roadway, and the VHMPC switches to the short prediction horizon when the vehicle is traveling on the continuous speed bump condition. Frequency sensors accomplish the acquisition of frequency information for continuous speed bump pavements.

**Figure 15:**
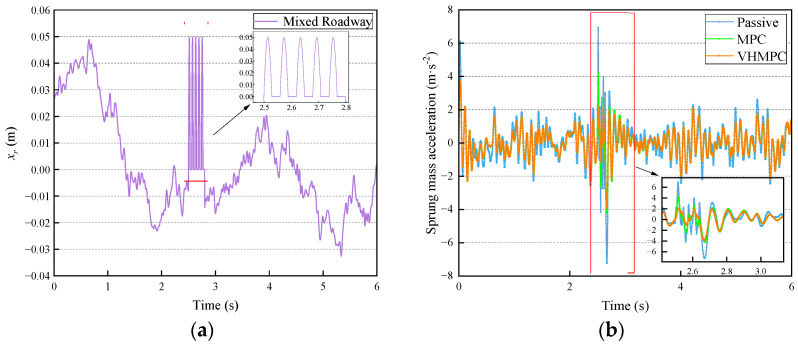

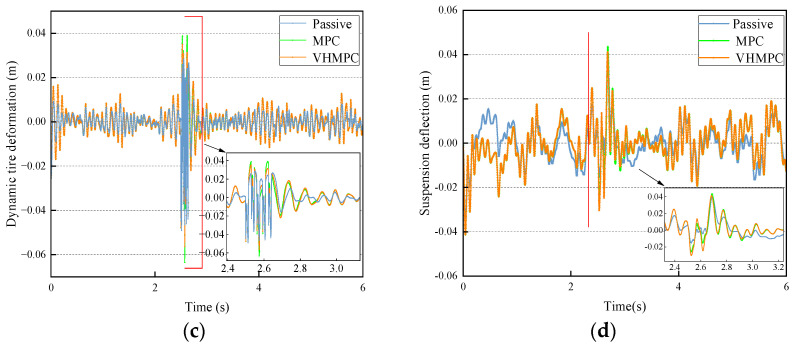
Time domain comparison of VHMPC suspension performance indexes for mixed roadway. (**a**) Mixed road excitation input. (**b**) Sprung mass acceleration. (**c**) Dynamic tire deformation. (**d**) Suspension deflection.

The braking deceleration process of the vehicle in front of the speed bump is not considered in the simulation. The passive suspension system (passive), traditional MPC, and VHMPC suspension system are simulated under the same road conditions. The comparison curves of sprung mass acceleration, dynamic tire deformation, and suspension deflection in the time domain for each type of suspension are shown in Figure 15. Their statistical properties are shown in Table 3.

**Figure 16:**
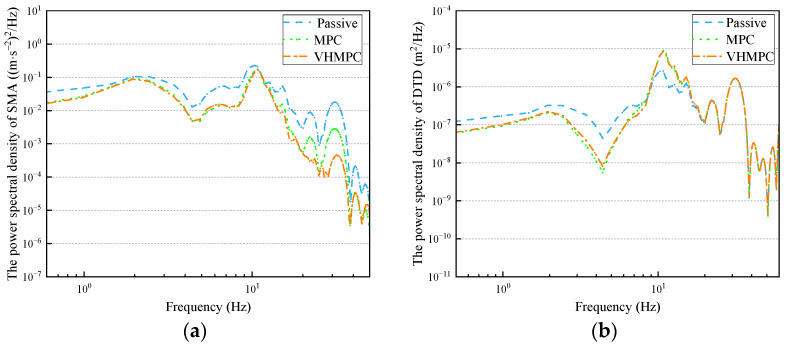

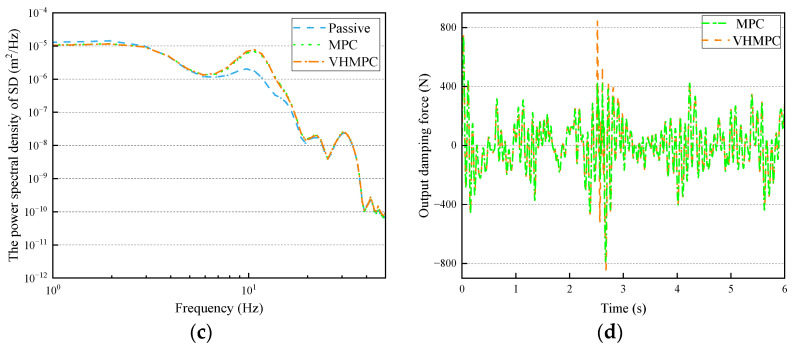
Frequency domain comparison of VHMPC suspension performance indexes for mixed roadway. (**a**) Sprung mass acceleration (SMA). (**b**) Dynamic tire deformation (DTD). (**c**) Suspension deflection (SD). (**d**) Output damping force.

As can be seen from Figure 16a, under mixed conditions, the sprung mass acceleration of VHMPC suspension has the lowest power spectral density in the low-frequency, medium-frequency, and high-frequency regions. As a result, the ride comfort of the VHMPC suspension is better than that of the MPC suspension and passive suspension. The VHMPC suspension is superior to MPC suspension and passive suspension in terms of power spectral density of dynamic tire deformation in the low-frequency and medium-frequency regions. In the high-frequency resonance region, the power spectral density of dynamic tire deformation and suspension deflection is worse for VHMPC suspension and MPC suspension than for passive suspension.

The power spectral density of dynamic tire deformation and suspension deflection in the high-frequency resonance region, MPC suspension, and VHMPC suspension are worse than passive suspension. This is because among the three evaluation indicators of the suspension system, it is difficult to optimize the three indicators to the best at the same time. The sprung mass acceleration power spectral density of VHMPC suspension is better than MPC suspension and passive suspension, which has a great improvement on ride comfort. While suspension deflection and dynamic tire deformation worsened, they are all within safe limits.

From the data in Figure 15 and Table 3, it can be seen that compared with passive suspension, MPC has a significant improvement in sprung mass acceleration (SMA), which can improve the smoothness of the vehicle by 23.449%, and VHMPC has a significant improvement on sprung mass acceleration, which can improve the smoothness by 25.451%; the two types of MPC under actual conditions have an improvement on the smoothness of vehicle driving under the actual conditions, both MPCs have improved the smoothness of the vehicle, indicating the effectiveness of the MPC to improve the smoothness of the vehicle; VHMPC suspension vehicle smoothness is the best, VHMPC relative to the MPC vehicle smoothness improved by 2.614%, in the discrete impact road (such as speed bumps) conditions, the sprung mass acceleration of the positive peak decreased by 11.849%, the negative peak decreased by 6.938%. The impact on passengers is significantly reduced, and the ride comfort is greatly improved.

The RMS value of the dynamic tire deformation of VHMPC suspension is the same as that of MPC suspension; the positive and negative peak ranges of dynamic tire deformation of VHMPC are reduced by 11.428% and 12.765%, respectively, relative to MPC, which shows that VHMPC’s driving safety is higher than that of MPC.

The root mean square (RMS) value of the suspension deflection of VHMPC is the same as that of the MPC suspension. Both of them deteriorate relative to the passive suspension, but neither of them exceeds the limiting travel of the suspension, which does not have much effect on the suspension performance; the positive peak range of suspension deflection for VHMPC decreased by 5.504% relative to that of MPC, and the negative peak is the same.

### 5.2. Variable Speed Conditions

**Figure 17:**
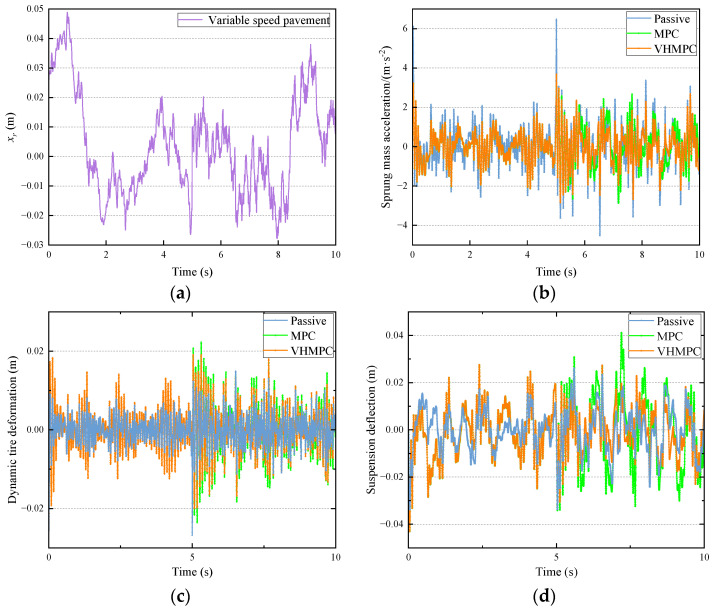
Time domain comparison of VHMPC suspension performance indexes for variable speed pavement. (**a**) Variable speed pavement excitation input. (**b**) Sprung mass acceleration. (**c**) Dynamic tire deformation. (**d**) Suspension deflection.

In the course of daily driving, a vehicle’s speed often changes abruptly. This can affect the suspension system, as the vehicle’s frequency of contact with the road changes. To further validate the effectiveness of VHMPC. This paper establishes a variable speed pavement model to test the control effect of VHMPC in vehicle variable speed conditions. The variable speed pavement model includes a total of 10 s of pavement excitation in the time domain. The first 5 s of pavement excitation is the vehicle driving at a speed of 10 m/s on a standard C class random pavement. The second 5 s of pavement excitation is the vehicle driving at a speed of 20 m/s on a standard C-class random pavement. The road surface input to the suspension excitation frequency is collected by the sensor, and the VHMPC automatically adjusts the level of the prediction horizon to match the level of the road surface excitation frequency. The vehicle travels at 10 m/s in the long prediction horizon state, and at 20 m/s in the short prediction horizon state. The acceleration process of the vehicle is not considered in the simulation. The simulation results are shown in Figure 17.

**Figure 18:**
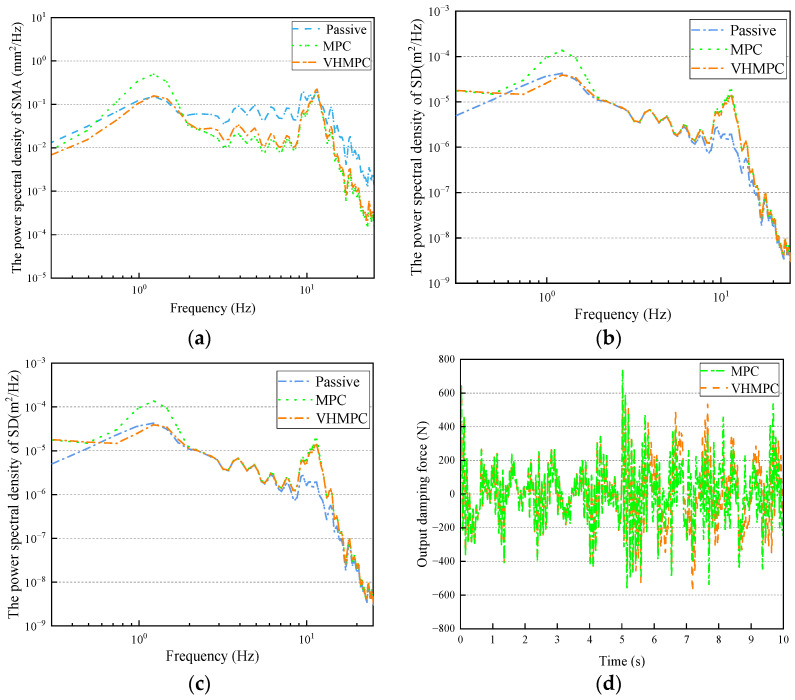
Frequency domain comparison of VHMPC suspension performance indexes for variable speed pavement. (**a**) Sprung mass acceleration (SMA). (**b**) Dynamic tire deformation (DTD). (**c**) Suspension deflection (SD). (**d**) Output damping force.

As can be seen from Figure 18, under variable speed conditions, in the low-frequency region, VHMPC suspension is superior to MPC suspension and passive suspension in terms of sprung mass acceleration and dynamic tire deformation power spectral density. In terms of the power spectral density of suspension deflection, VHMPC suspension is better than MPC suspension, but it is worse than passive suspension. In the intermediate frequency region, the power spectral density of sprung mass acceleration and dynamic tire deformation is better than that of passive suspension by VHMPC suspension, but slightly worse than MPC suspension. In the high-frequency resonance region, the power spectral density of dynamic tire deformation and suspension deflection is worse for VHMPC suspension and MPC suspension than for passive suspension.

The sprung mass acceleration power spectral density of VHMPC suspension has a better performance among the three suspension types, which indicates that VHMPC suspension can effectively improve ride comfort. Although there is a slight deterioration in the power spectral density of dynamic tire deformation and suspension deflection, this is because the performance indicators of the three suspensions are difficult to achieve simultaneous optimization, and the deterioration is within the range of safety.

From the data in Figure 17 and Table 4, it can be seen that in terms of sprung mass acceleration, the MPC and VHMPC are improved by 19.058% and 24.878%, respectively, compared with passive suspension. VHMPC outperforms MPC by 7.191% in terms of sprung mass acceleration, 7.936% in terms of dynamic tire deformation, and 22.222% in terms of suspension deflection. This proves that VHMPC is a superior suspension control method to traditional MPC.

## 6. Conclusions

(1) The dynamics of the magnetorheological damper are tested and analyzed by establishing a dynamics performance test system for the magnetorheological damper, and the forward and inverse models of the magnetorheological damper are established based on the experimental data. The improved hyperbolic tangent model is chosen as the forward model of the magnetorheological damper. The particle swarm algorithm is used to identify the parameters to be identified in the model, and a comparative analysis is made between the experimental data and the identified model; at the same time, the ANFIS inverse model of the magnetorheological damper is proposed to be used for the solution of the required current of the magnetorheological damper. The simulation results show that the inverse model has a small inference error and can be used in the magnetorheological semi-active suspension with air springs.
(2) By analyzing the relationship between the model prediction control effect and road excitation frequency under different prediction horizon parameters, it is found that the prediction horizon which makes the suspension damping effect the best is negatively correlated with the road excitation frequency, indicating that different prediction horizons should be set for different road excitation frequencies.
(3) Under the mixed continuous speed bump condition, the VHMPC suspension improves the sprung mass acceleration by 2.614% relative to the conventional MPC suspension, and the positive and negative peaks of the sprung mass acceleration decrease by 11.849% and 6.938%, respectively. The VHMPC suspension has the same dynamic tire deformation as the conventional MPC suspension, but the positive and negative peaks of the dynamic tire deformation decrease by 11.428% and 12.765%. Under changing speed pavement conditions, the MPC suspension and VHMPC suspension improve the sprung mass acceleration by 19.058% and 24.878%, respectively, compared to the passive suspension, and the VHMPC suspension improves by 7.191% relative to the MPC suspension. VHMPC suspension improves the dynamic tire deformation by 7.936% and the suspension deflection by 22.222% compared to the MPC suspension.

There are some shortcomings in this study, such as not considering the time response of magnetorheological damper. In the follow-up research plan, we will further verify the effectiveness of the VHMPC algorithm in a real environment.

## Figures and Tables

**Table 1:** Identification parameter values.

Parameter	$a_{1}$	$a_{2}$	k	$a_{3}$	$f_{0}$
Current					
0 A	33.42	59.65	0.8000	314.05	−3.2
0.25 A	155.25	75.74	0.7278	804.51	−6.28
0.5 A	371.87	41.50	1.1298	1130.89	−8.84
0.75 A	496.86	40.65	1.3871	1503.23	−9.79
1 A	581.21	34.34	1.4984	1801.39	−9.17

**Table 2:** Parameters of air spring.

Air Pressure (MPa)	Outer Diameter (mm)	Load (KN)
0.1	150	1.1
0.3	160	3.5
0.5	164	6.1
1	164	13.5

**Table 3:** Statistical characteristics of response quantities of vibration characteristics of suspension system.

Suspension Systems	Sprung Mass Acceleration/(m·s^−2^)		Dynamic Tire Deformation/m		Suspension Deflection/m	
	RMS	Peak Range	RMS	Peak Range	RMS	Peak Range
Passive	1.0593	[−7.2511, 6.9473]	0.0046	[−0.0491, 0.0283]	0.0082	[−0.0339, 0.0398]
MPC	0.8109	[−4.2173, 4.2130]	0.0057	[−0.0636, 0.0390]	0.0091	[−0.0415, 0.0436]
VHMPC	0.7897	[−3.9247, 3.7138]	0.0057	[−0.0564, 0.0350]	0.0091	[−0.0415, 0.0412]

**Table 4:** Statistical characteristics of response quantities of vibration characteristics of suspension.

Suspension Systems	RMS		
	Sprung Mass Acceleration/(m·s^−2^)	Dynamic Tire Deformation/m	Suspension Deflection/m
Passive	1.1717	0.0039	0.0092
MPC	0.9484	0.0063	0.0135
VHMPC	0.8802	0.0058	0.0105

## Data Availability

Data are contained within the article.
